# Aging‐associated reduction of chromosomal histones in mammalian oocytes

**DOI:** 10.1111/gtc.13146

**Published:** 2024-07-23

**Authors:** Masashi Mori, Manami Koshiguchi, Osamu Takenouchi, Mei A. Mukose, Hinako M. Takase, Tappei Mishina, Hailiang Mei, Miho Kihara, Takaya Abe, Azusa Inoue, Tomoya S. Kitajima

**Affiliations:** ^1^ Laboratory for Chromosome Segregation RIKEN Center for Biosystems Dynamics Research (BDR) Kobe Japan; ^2^ Graduate School of Biostudies Kyoto University Kyoto Japan; ^3^ Laboratory for Animal Resources and Genetic Engineering RIKEN Center for Biosystems Dynamics Research (BDR) Kobe Japan; ^4^ Laboratory for Epigenome Inheritance, RIKEN Center for Integrative Medical Sciences Yokohama Japan; ^5^ Present address: Faculty of Agriculture Kyushu University Fukuoka Japan

**Keywords:** aging, chromosome, meiosis, oocyte

## Abstract

Mammalian oocytes undergo a long‐term meiotic arrest that can last for almost the entire reproductive lifespan. This arrest occurs after DNA replication and is prolonged with age, which poses a challenge to oocytes in maintaining replication‐dependent chromosomal proteins required for the completion of meiosis. In this study, we show that chromosomal histones are reduced with age in mouse oocytes. Both types of histone H3 variants, replication‐dependent H3.1/H3.2 and replication‐independent H3.3, decrease with age. Aging‐associated histone reduction is associated with transcriptomic features that are caused by genetic depletion of histone H3.3. Neither the genetic reduction of chromosomal H3.1/H3.2 nor H3.3 accelerates the aging‐associated increase in premature chromosome separation that causes meiotic segregation errors. We suggest that aging‐associated reduction of chromosomal histones is linked to several transcriptomic abnormalities but does not significantly contribute to errors in meiotic chromosome segregation during the reproductive lifespan of mice.

## Graphical abstract

Mammalian oocytes undergo a long‐term cell cycle arrest following premeiotic DNA replication. This study shows that chromosomal histones, including replication‐dependent H3.1/H3.2 and replication‐independent H3.3, decrease with age.

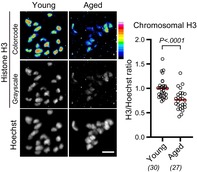

## 1 INTRODUCTION

In most mammals including humans, oocytes, the female germ cells, are produced only during fetal development and enter a prolonged cell cycle arrest known as dictyate arrest (Charalambous et al., 2023; Herbert et al., 2015; MacLennan et al., 2015). This cell cycle arrest, initiated after premeiotic S‐phase and meiotic recombination at the fetal stage, persists until meiotic resumption, which transitions to the M‐phase of meiosis before ovulation at an adult stage. Thus, dictyate arrest can span much of the female reproductive lifespan, lasting months in mice and decades in humans. The prolonged duration of dictyate arrest presents a challenge to oocytes in maintaining the machinery required for the proper completion of meiosis. One of the critical components of the machinery is chromosome cohesion, which is established by the protein complex cohesin through a mechanism coupled to DNA replication in fetal oocytes (Burkhardt et al., 2016). During the subsequent dictyate arrest, due to the lack of DNA replication, oocytes are unable to replenish cohesin on chromosomes (Burkhardt et al., 2016), and thus chromosomal cohesin gradually decreases with age in mouse oocytes (Chiang et al., 2010; Lister et al., 2010). As a result, aged oocytes exhibit weakened chromosome cohesion, which facilitates chromosome segregation errors during the M‐phase of meiosis (Sakakibara et al., 2015; Zielinska et al., 2015).

In addition to the establishment of chromosome cohesion, DNA replication‐dependent chromosomal processes include the deposition of histone H3 variants H3.1 and H3.2, mediated by their specific chaperone complex CAF1 (Stewart‐Morgan et al., 2020). As histone‐DNA nucleosomes are responsible for the structural and functional integrity of chromosomes, oocytes must maintain chromosomal histones during the long period of dictyate arrest without DNA replication. This maintenance is facilitated by the replication‐independent deposition of the histone H3 variant H3.3 via its specific chaperone complex containing HIRA (Nashun et al., 2015). Deletion of the *Hira* gene in mouse oocytes before dictyate arrest results in reduced levels of chromosomal histones, accompanied by various chromatin defects, including transcriptional dysregulation, leading to oocyte depletion by 1–2 months after birth (Nashun et al., 2015). Whether the replication‐independent mechanism maintains chromosomal histones at a constant level in oocytes throughout life (>1 year in mice) remains unclear.

Here, we perform an immunofluorescence assay on condensed chromosomes in mouse oocytes at M‐phase of meiosis I (MI) to quantify chromosomal histones at different ages. This analysis shows that chromosomal histones, including both replication‐dependent H3.1/H3.2 and replication‐independent H3.3, are reduced on chromosomes with age. Transcriptome analysis of aged oocytes reveals several characteristic transcriptomic features that resemble those induced by conditional deletion of *Hira*. We establish mouse genetic models that specifically manipulate the levels of chromosomal H3.1/H3.2 and H3.3 by generating floxed alleles of genes for CAF1 and HIRA, respectively. Live imaging of oocytes from these models at high resolution shows that genetic reduction of H3.1/H3.2 or H3.3 does not accelerate the aging‐associated increase in premature chromosome separation, a major cause of chromosome segregation errors during meiosis in aged oocytes.

## 2 RESULTS

### 2.1 Reduction of chromosomal histones in oocytes with age

**FIGURE 1:**
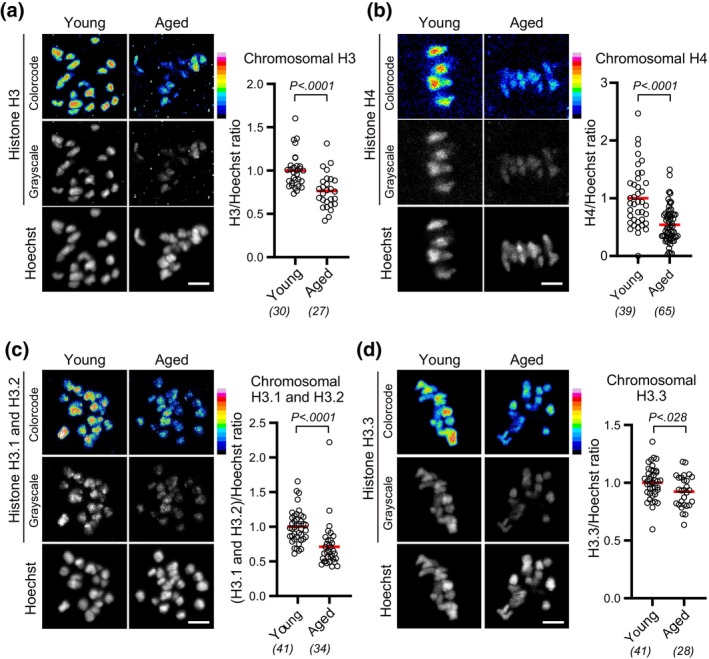
Chromosomal H3 and H4 reduce with age. (a–d) Aging‐associated reduction in chromosomal histone H3 and H4. BDF1 oocytes at MI (meiosis I) were immunostained with anti‐H3 (a), anti‐H4 (b), anti‐H3.1/H3.2 (c), or anti‐H3.3 (d), and Hoechst33342. Color code with 16 colors. Mice at 2 months old (young) and 14–22 months old (aged) were used. Intensity of immunofluorescence relative to that of Hoechst33342 is shown. Mann–Whitney test. Parentheses show the number of oocytes from 3 (a), 2 (b), or 4 (c,d) independent experiments. Scale bar, 5 μm.

To investigate aging‐associated changes in chromosomal histones, we obtained oocytes from young (2 months old) and aged (14–22 months old) BDF1 mice and immunostained them with H3 and H4 antibodies at MI. Quantification of chromosomal fluorescence signals revealed that aged oocytes carried significantly reduced levels of chromosomal H3 and H4 compared with young oocytes (Figure 1a,b). In contrast, the levels of chromosomal H2A or H2B were not significantly reduced (Figure S1A,B). Reduction of H3 and H4 with intact levels of H2A and H2B is reported in oocytes conditionally deleted of the *Hira* gene (Nashun et al., 2015). To examine whether either or both H3 variants, replication‐dependent H3.1/H3.2 and replication‐independent H3.3, are reduced with age, we immunostained oocytes with their specific antibodies. Quantification showed that both chromosomal H3.1/H3.2 and H3.3 were significantly reduced in aged oocytes (Figure 1c,d). We observed similar reductions in chromosomal H3 variants in aged oocytes from C57BL/6 mice (Figure S1C,D). These results suggest that chromosomal H3‐H4, regardless of whether replication‐dependent or ‐independent variants, are reduced with age, although the extent of nucleosomal histone reduction is unknown.

### 2.2 Transcriptomic features related to histone loss in aged oocytes

**FIGURE 2:**
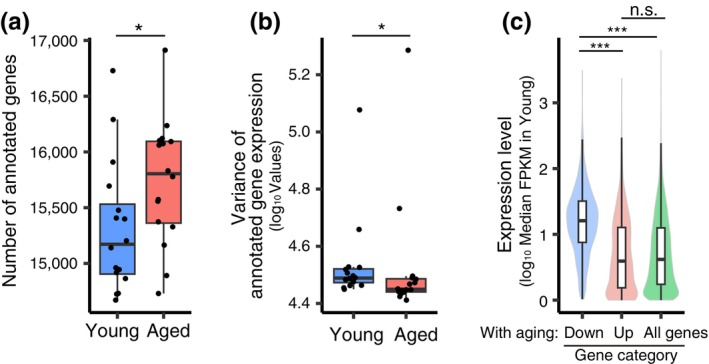
Transcriptomic features of aged mice. (a) Number of annotated genes (CPM >1) from young and aged oocytes using RamDA‐seq. *: *P* = .047 using Welch's *t*‐test. (b) Log10‐scaled variance of gene expression among all annotated genes of each oocyte. *: *P* = .015 using Wilcoxon's rank‐sum test. (c) Violin plot showing the relationships between the distribution of median expression levels (FPKM) in young oocytes and the DEGs between young and aged oocytes. Significant differences between categories were tested using Welch's *t*‐test with Holm's correction for multiple comparisons. ***: Aged down versus aged up: *P* = 4.8e−6; aged down versus all genes: *P* = 9.1e−12; n.s.: Aged up versus all genes: *P* = .26.

We investigated whether the aging‐associated reduction in chromosomal H3–H4 is accompanied by chromatin and transcriptome changes that are caused by histone loss. The previous study showed that in oocytes conditionally deleted of *Hira*, a global loss of chromosomal H3–H4 is associated with a limited capacity to fine‐tune gene expression levels throughout the genome (Nashun et al., 2015). To examine the global nucleosomal status, we performed micrococcal nuclease (MNase)‐seq analysis (Hughes & Rando, 2014) with the MI chromosomes of conditional *Hira* knockout oocytes, which were obtained by crossing a newly established floxed *Hira* allele (Figure S2) with the oocyte‐specific *Gdf9*‐Cre recombinase (Lan et al., 2004). *Gdf9*‐Cre is activated around premeiotic S‐phase, a stage before dictyate arrest, and deletes a target gene with 90% efficiency (Burkhardt et al., 2016). MNase‐seq analysis showed that the nucleosomal pattern at promotors in conditional *Hira* knockout oocytes was less distinct than in control oocytes (Figure S3A,B), confirming their severe defects in chromatin integrity (Nashun et al., 2015). In contrast, the nucleosomal pattern in aged oocytes was not less distinct than in young oocytes (Figure S3A,B), consistent with the idea that aged oocytes carry largely maintained nucleosomal histones. However, transcriptome analysis of our published datasets of aged oocytes (Mishina et al., 2021) revealed a significantly increased number of transcribed genes (Figure 2a), suggesting a defect in gene silencing, similar to conditional *Hira* knockout oocytes (Nashun et al., 2015). Furthermore, we detected a significantly decreased variance in expression levels among transcribed genes in aged oocytes (Figure 2b), suggesting a limited dynamic range in controlling expression levels, as observed in conditional *Hira* knockout oocytes (Nashun et al., 2015). Consistently, we found that genes that were down‐regulated with aging tend to be the ones that were highly expressed in young oocytes (Figure 2c). Thus, several transcriptomic features of aged oocytes are consistent with those caused by histone loss, to which aging can be a contributing factor.

### 2.3 Chromosome segregation abnormalities caused by histone loss are distinct from those associated with aging

**FIGURE 3:**
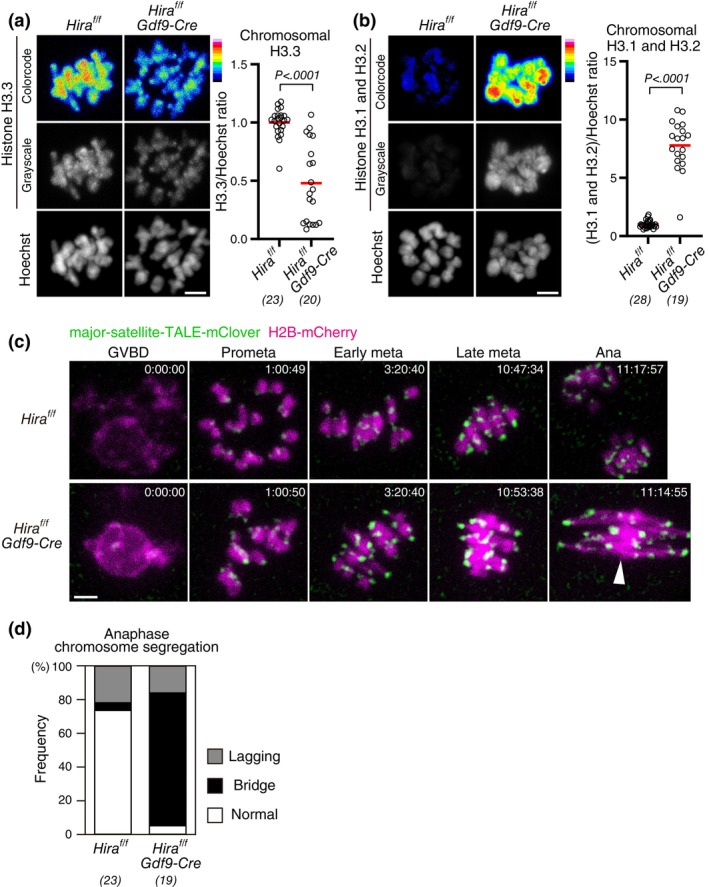
Conditional deletion of *Hira* results in chromosomal abnormalities distinct from those of aged oocytes. (a,b) Conditional *Hira* deletion results in decreased H3.3 and increased H3.1/H3.2. Oocytes at MI were immunostained with anti‐H3.3 (a) or anti‐H3.1/H3.2 (b) and Hoechst33342. Color code with 16 colors. BDF1 mice at 2 months old (young) and 14–22 months old (aged) were used. Intensity of immunofluorescence relative to that of Hoechst33342 is shown. Mann–Whitney test. Parentheses show the number of oocytes from three independent experiments. Scale bar, 5 μm. (c,d) Conditional *Hira knockout* oocytes exhibit anaphase bridges. Oocytes collected from mice at 4 weeks old were monitored for MI with major‐satellite‐TALE‐mClover (centromere) and H2B‐mCherry (chromosome). Centromere signals were processed for peak enhancement and background subtraction. Time after germinal envelope breakdown (GVBD, entry to M‐phase) is shown (h:mm:ss). The arrowhead indicates the anaphase bridge. Scale bar, 5 μm. In (d), oocytes that underwent anaphase were categorized based on anaphase figures. Parentheses show the number of anaphases from six independent experiments.

Next, we aimed to determine whether the reduction of chromosomal H3–H4 contributes to aging‐associated chromosome segregation errors in oocytes. We speculated that conditional *Hira* knockout oocytes could be a good, although not perfect, model to address this question. To characterize the chromosomal H3 composition of conditional *Hira* knockout oocytes, we immunostained them with a specific H3.3 antibody, which confirmed that conditional *Hira* knockout oocytes carried a significantly decreased chromosomal H3.3 (Figure 3a). In contrast, immunostaining with a H3.1/H3.2‐specific antibody showed a significantly increased level of chromosomal H3.1/H3.2 in conditional *Hira* knockout oocytes (Figure 3b). This increased H3.1/H3.2 is likely a fraction that persisted abnormally after DNA replication due to the absence of HIRA, as HIRA normally replaces H3.1/H3.2 with H3.3 on chromosomes after DNA replication (Ahmad & Henikoff, 2002; Tagami et al., 2004). Thus, conditional *Hira* knockout oocytes are a model for oocytes with reduced H3.3 and increased H3.1/H3.2.

We acquired 4D datasets of chromosome dynamics with the chromosome marker H2B‐mCherry and the centromere marker major‐satellite‐TALE‐mClover (Miyanari et al., 2013) in conditional *Hira* knockout oocytes throughout meiosis I using live confocal microscopy (Figure 3c). We performed this at a high spatiotemporal resolution capable of detecting major aging‐associated defects, such as premature chromosome separation (Kitajima et al., 2011; Sakakibara et al., 2015). We found that conditional *Hira* knockout oocytes frequently exhibited anaphase bridges (Figure 3c,d), consistent with the previous report (Nashun et al., 2015), and different from aged oocytes, where anaphase bridges are not increased (Sakakibara et al., 2015). Despite the highly frequent anaphase defects in conditional *Hira* knockout oocytes, their prometaphase or metaphase did not exhibit premature chromosome separation (0 of 58 oocytes), a major event that precedes anaphase segregation errors in aged oocytes (Sakakibara et al., 2015). These results show that conditional *Hira* knockout oocytes result in chromosome segregation abnormalities that are distinct from those caused by aging‐associated defects.

### 2.4 *Hira* insufficiency does not accelerate aging‐associated increase in chromosome segregation errors

**FIGURE 4:**
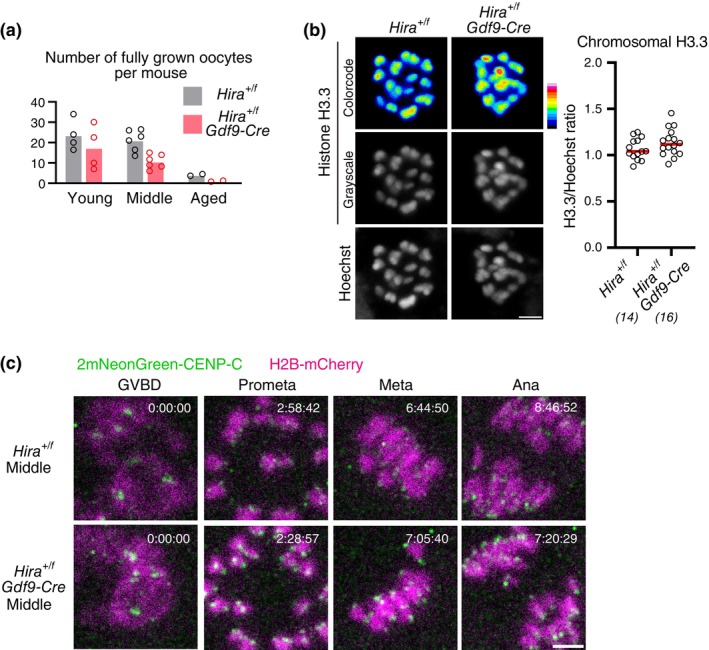
Conditional heterozygous deletion of *Hira* does not accelerate aging‐associated increase in premature chromosome separation. (a) Conditional heterozygous *Hira* knockout accelerates a decrease in the number of oocytes. Fully grown oocytes were collected from the ovaries of mice at 2–4 months old (young), 6–8 months old (middle), and 12–15 months old (aged). Dots indicated data from individual mice. 5 (young), 6 (middle) and 2 (aged) independent experiments were performed. (b) Conditional heterozygous *Hira* knockout results in undetectable decrease in H3.3. Oocytes at MI were immunostained with anti‐H3.3 and Hoechst33342. Color code with 16 colors. Mice at 2 months old were used. Intensity of immunofluorescence relative to that of Hoechst33342 is shown. Mann–Whitney test. Parentheses show the number of oocytes from two independent experiments. Scale bar, 5 μm. (c) Conditional heterozygous *Hira* knockout does not exhibit increased premature chromosome separation. Oocytes collected from mice at 2–4 months old (young) and at 6–8 months old (middle) were monitored for MI with 2mNeonGreen‐CENP‐C (centromere) and H2B‐mCherry (chromosome). Premature chromosome separation was observed in 1/86 oocytes from *Hira* ^*+/f*^ mice and 0/71 oocytes from *Hira* ^*+/f*^ *Gdf9‐Cre* mice at the middle age; and in 1/76 oocytes from *Hira* ^*+/f*^ mice and 1/66 *Hira* ^*+/f*^ *Gdf9‐Cre* mice at the young age. Six independent experiments were performed. Centromere signals were processed for peak enhancement and background subtraction. Time after GVBD is shown (h:mm:ss). Scale bar, 5 μm.

It is possible that histone loss due to conditional *Hira* knockout causes inefficient chromosome condensation. Consistently, anaphase bridges found in conditional *Hira* knockout oocytes resembled those observed in oocytes deficient in chromosome condensation due to condensin dysfunction (Houlard et al., 2015; Lee et al., 2011). Insufficient condensation can lead to chromosome entanglement, which may have prevented premature chromosome separation in conditional *Hira* knockout oocytes. We therefore asked whether a mild loss of chromosomal H3.3–H4, as observed in aged oocytes, could facilitate premature chromosome separation. If so, conditional heterozygous *Hira* knockout (*Hira* ^*+/flox*^ *Gdf9‐Cre*) could accelerate the aging‐associated increase in premature chromosome separation in oocytes. Conditional heterozygous *Hira* knockout accelerated aging‐associated reduction of fully grown oocytes in ovaries (Figure 4a), while their chromosomal H3.3 levels were not significantly different from those of control oocytes (Figure 4b). These observations are consistent with the idea that conditional heterozygous *Hira* knockout oocytes carry a slightly and undetectably reduced amount of chromosomal histones. We then performed live imaging of conditional heterozygous *Hira* knockout oocytes at young (2–4 months old) and middle (6–8 months old) ages (Figure 4c), although we could not analyze oocytes from aged mice (12–15 months old) due to their severe scarcity (Figure 4a). This analysis showed that *Hira* insufficiency caused no detectable increase in premature chromosome separation at the middle age (1/86 oocytes in *Hira* ^*+/f*^ vs. 0/71 oocytes in *Hira* ^*+/f*^ *Gdf9‐Cre*). These data do not provide evidence for a link between reduced chromosomal H3.3 and aging‐associated chromosome segregation errors in oocytes.

### 2.5 Chromosomal H3.1/H3.2 insufficiency does not accelerate aging‐associated increase in chromosome segregation errors

**FIGURE 5:**
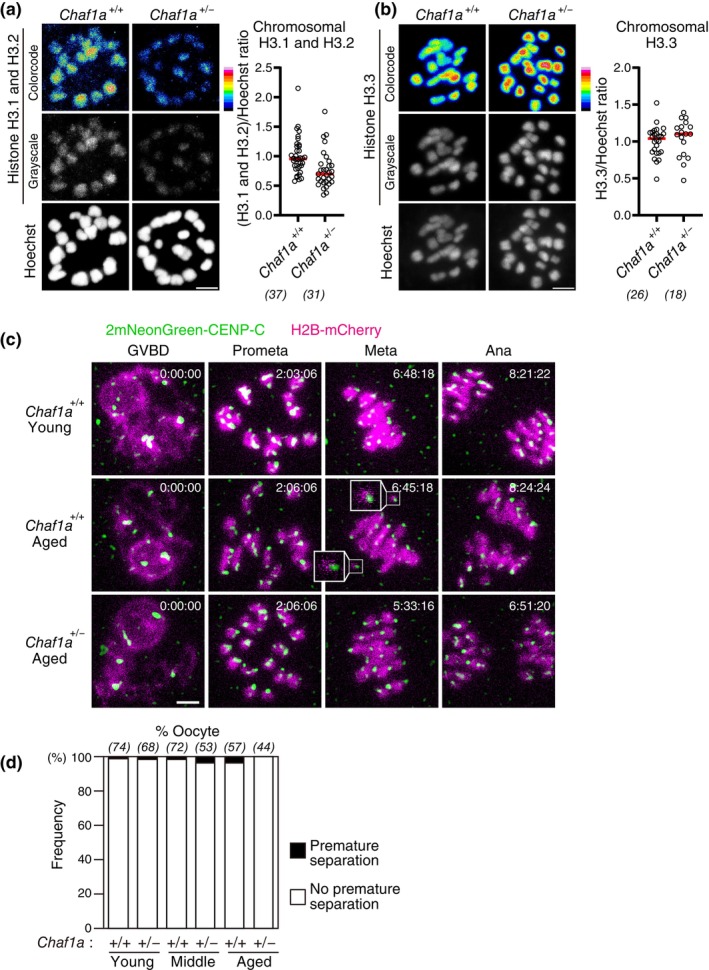
Heterozygous deletion of *Chaf1a* does not accelerate aging‐associated increase in premature chromosome separation. (a,b) Heterozygous *Chaf1a* knockout results in a reduction in H3.1/H3.2 but not in H3.3. Oocytes at MI were immunostained with anti‐H3.1/2 (a) or anti‐H3.3 (b) and Hoechst33342. Color code with 16 colors. Mice at 4–8 months old were used. Intensity of immunofluorescence relative to that of Hoechst33342 is shown. Mann–Whitney test. Numbers in parentheses show oocytes from four independent experiments. Scale bar, 5 μm. (c,d) Heterozygous *Chaf1a* knockout does not exhibit increased premature chromosome separation. Oocytes collected from mice at 3–4 months old (young), 6–8 months old (middle), and 12–15 months old (aged) were monitored for MI with 2mNeonGreen‐CENP‐C (centromere) and H2B‐mCherry (chromosome). Centromere signals were processed for peak enhancement and background subtraction. Insets show prematurely separated chromosomes. Time after GVBD is shown (h:mm:ss). Scale bar, 5 μm. In (d), the frequency of oocytes that exhibited premature chromosome separation is shown. Parentheses show the number of oocytes from 4 (young, middle) or 8 (aged) independent experiments.

We next addressed whether a reduction of H3.1/H3.2, but not H3.3, facilitates chromosome segregation errors. To test this idea, we generated a null knockout allele of *Chaf1a* (Figure S4), the gene encoding a subunit of the H3.1/H3.2‐specific histone chaperone complex CAF1 (Stewart‐Morgan et al., 2020). As expected, heterozygous *Chaf1a* knockout oocytes had a reduced level of chromosomal H3.1/H3.2 to 76% ± 31% (Figure 5a), a level comparable to aged oocytes (Figure 1c), while exhibiting an intact level of H3.3 (Figure 5b). Live imaging of heterozygous *Chaf1a* knockout oocytes from young (3–4 months old), middle (6–8 months old), and aged (12–15 months old) mice showed no detectable accelerated increase in premature chromosome separation (Figure 5c,d). These data suggest that reduced chromosomal H3.1/H3.2 does not significantly contribute to aging‐associated chromosome segregation errors.

## 3 DISCUSSION

Oocyte dictyate arrest, which can span almost the entire reproductive lifespan, poses a significant challenge to maintaining structural and functional integrity over a long period of time, ranging months in mice and decades in humans. Dictyate arrest lacks DNA replication, which is required for a number of proteins to be deposited on chromosomes, and thus puts replication‐dependent chromosomal proteins at risk of aging‐dependent degradation or dysfunction. This study shows that both replication‐dependent and ‐independent histone H3 variants, H3.1/H3.2 and H3.3, respectively, decrease on chromosomes with age. Although this aging‐associated reduction in chromosomal histones does not appear to be associated with an extensive disruption of nucleosomal profiles, it is associated with several transcriptomic features caused by genetic depletion of chromosomal histones. However, genetic reduction of H3.1/H3.2 or H3.3 did not accelerate the increase in chromosomal defects that cause chromosome segregation errors in aged oocytes, unlike genetic cohesion reduction that causes a significant acceleration (Hodges et al., 2005). Although these results provide insights into potential effects of aging‐associated H3 reduction on oocyte integrity, it should be noted that our study has several limitations. First, the conditional homozygous or heterozygous *Hira* knockout or heterozygous *Chaf1a* knockout oocytes used in this study do not show reductions in both H3.1/2 and H3.3 on chromosomes, allowing us to only partially mimic aging‐associated histone reduction. Second, the frequency of premature chromosome separation in aged wild‐type C57BL/6 oocytes (4% in *Chaf1a* ^*+/+*^ oocytes at 12–15 months old, Figure 5d) did not appear to be as high as that reported in aged BDF1 oocytes (9.5% at 16 months old) (Sakakibara et al., 2015), suggesting a possibility that the strain background used in this study did not provide a sensitive assay for potential acceleration of aging‐associated errors. Nevertheless, the results obtained from the genetic models used in this study are consistent with the idea that aging‐associated reduction in chromosomal histones is linked to several transcriptomic features in aged oocytes but does not significantly contribute to chromosome segregation errors during the normal reproductive life in mice. How chromosomal histones are maintained in oocytes over the much longer reproductive lifespan of decades in humans is an interesting question for future studies.

The aging‐associated decrease in H3.1/H3.2 is consistent with gradual replacement by H3.3 through the action of the DNA replication‐independent chaperone containing HIRA (Nashun et al., 2015). At 4 weeks after birth, the level of chromosomal H3.1/H3.2 in control oocytes was ~12% of that in conditional *Hira* knockout oocytes (Figure 3b), suggesting that the majority of H3.1/H3.2 is rapidly removed from chromosomes in a HIRA‐dependent manner during the juvenile period. The replacement of chromosomal H3.1/H3.2 by H3.3 is coupled with the establishment of the non‐canonical pattern of H3.3 across the oocyte genome, which may contribute to the acquisition of a global chromatin status that allows the earliest transcriptional program after fertilization (Ishiuchi et al., 2021). It is possible that abnormally retained H3.1/H3.2, as well as reduced H3.3, contributes to developmental defects observed in embryos derived from conditional *Hira* knockout oocytes (Nashun et al., 2015; Smith et al., 2022). In contrast to the rapid H3.1/H3.2 removal during the juvenile period, the rate of H3.1/H3.2 reduction during the adult period appears to be slower, because aged oocytes (>10 months old) retained ~71% of H3.1/H3.2 compared with young oocytes (2 months old) (Figure 1c). Some fractions of chromosomal H3.1/H3.2 may not be subject to replication‐independent histone turnover through replacement by H3.3.

In contrast to the aging‐associated reduction in chromosomal H3.1/H3.2, the reduction in chromosomal H3.3 cannot be explained by the absence of DNA replication. Aging may affect the expression levels of genes that contribute to HIRA‐mediated H3.3 turnover, perhaps with gradual exhaustion of replication‐dependent chromosomal factors. Alternatively, HIRA itself may undergo aging‐associated dysfunction or degradation, as HIRA is extremely long‐lived and undergoes little turnover in oocytes (Harasimov et al., 2024). We found several transcriptomic features of aged oocytes, including a limited dynamic range of gene expressions, which resemble those of conditional *Hira* knockout oocytes, although a causal relationship of these transcriptomic features with aging‐associated reduction in chromosomal histones is unclear. Aging‐associated reduction in chromosomal histones is not associated with nucleosome profile abnormalities detectable by MNase‐seq. Nevertheless, the possibility that aging‐associated reduction in chromosomal histones contribute to fine‐tuning transcriptomic regulation cannot be excluded.

Global histone reduction on chromosomes is observed not only in mammalian oocytes, but also in various cell types across species, including human senescent cells (Pal & Tyler, 2016). In the brain, which contains post‐mitotic neurons, H3.1/H3.2 decreases while H3.3 accumulates with aging (Maze et al., 2015). Life‐long maintenance of chromosomal histones may be a common challenge for various cell types, and deterioration of the maintenance may be linked to organismal aging.

## 4 EXPERIMENTAL PROCEDURES

### 4.1 Mouse experiments

Genetically engineered mice were based on the C57BL/6 background. BDF1 mice were used in the experiments shown in Figures 1, 2, S1A,B, and S3 (young and aged). Mice were purchased from Japan SLC, Inc. *Gdf9‐*Cre mice were obtained from the Jackson Laboratory (Stock no. 0011062). All mouse experiments were approved by the Institutional Animal Care and Use Committee at RIKEN Kobe Branch (IACUC).

### 4.2 Genetic engineering

The *Hira* conditional knockout (floxed) mice (Accession No.: CDB0105E: https://large.riken.jp/distribution/mutant-list.html) and the *Chaf1a* floxed mice (Accession No.: CDB0106E) were generated by CRISPR/Cas9‐mediated genome editing in C57BL/6 zygotes using single‐strand oligodeoxynucleotides (ssODN) as previously described (Hashimoto et al., 2016). gRNA sites were designed using CRISPRdirect (Naito et al., 2014), and crRNA/tracrRNA and ssODN were chemically synthesized (Fasmac Co., Ltd). The floxed alleles are shown in Figures S2 and S4. Routine genotyping PCR was performed using following primers; 5gtFW (5′‐CAT CCG GCT TCA CCA TCC‐3′) and 5gtREV (5′‐TCT CCT GTG CAC CAC TGG‐3′) (WT: 289 bp, 5′‐loxP: 329 bp) and 3gtFW(5′‐ GGC CAT ATG ACA ACT AGC‐3′) and 3gtREV(5′‐TGC CTC TAC CTC CCA TGC‐3′) (WT: 267 bp, 3′‐loxP: 307 bp) for *Hira* floxed mice, and 5gtFW (5′‐CTC CTC TCT TAG TGC TGC‐3′) and 5gtREV (5′‐TGT GGG TCT CGT GGA GCC‐3′) (WT: 283 bp, 5′‐loxP: 323 bp), and 3gtFW (5′‐TTG TGA GCC ACC ATG TGG‐3′) and 3gtREV (5′‐GAC ACT GCC TCA GTA GGC‐3′) (WT: 188 bp, 3′‐loxP: 228 bp) for *Chaf1a* floxed mice.

### 4.3 Mouse oocytes

Fully grown oocytes were isolated from the ovaries 48 h after injection of pregnant mare's serum gonadotropin (Serotropin, ASKA Animal Health) into female mice, and cultured in M2 medium containing 3‐isobutyl‐1‐metyl‐xanthine (IBMX, Sigma). The mixture of mRNA was introduced by microinjection, and then oocytes were cultured for at least 2 h at 37°C. The oocytes were released from IBMX by washing to induce meiotic resumption.

### 4.4 Transcriptome analysis

We re‐analyzed our previously published dataset of single oocyte transcriptomes from young (2‐month‐old) and aged (12‐month‐old) BDF1 mice (GSE159281) (Mishina et al., 2021). The number of annotated genes with counts per million (CPM) >1 was counted for each oocyte, and their difference with respect to the mouse age class was tested using Welch's *t*‐test. The variance of gene expression of all annotated genes (CPM >0) in each oocyte was compared between the age classes using Wilcoxon's rank sum test because its distribution violated the assumption of Gaussian distribution (*P* = 1.32e−10 using Shapiro–Wilk test). Finally, the relationships between expression levels in oocytes of young mice and differential expression were examined. The list of differentially expressed genes (DEGs) reported in our previous study (Mishina et al., 2021) was used. Briefly, these DEGs (false discovery rate, FDR <0.05 calculated using the R package “*q*value”) were identified using the edgeR package (Robinson et al., 2010) in R, which normalizes library sizes with the trimmed mean of *M* values (TMM) method, using the dataset of genes expressed in at least five samples.

### 4.5 Low‐input MNase‐seq

Ten MI oocytes per replicate were sampled into a tube containing the lysis buffer, frozen with liquid nitrogen, and subjected to MNase‐seq as previously described (Sakamoto et al., 2023). The MNase treatment condition was 21°C for 7.5 min. The CAM pipeline (Hu et al., 2017) was used to assess the data quality of MNase‐seq. Paired‐end sequencing was performed on a Nextseq 2000. The sequencing read information was summarized in Table S1. The data processing was performed in accordance with (Sakamoto et al., 2023). The sequence data sets were deposited to the Sequence Read Archive (SRA) under accession number PRJNA1122508.

### 4.6 Immunostaining of oocytes

Oocytes were pre‐extracted in PBT (PBS with 0.1% Triton X‐100) for 1 min. The oocytes were fixed with 3.2% formaldehyde (methanol‐free) in PBT for 10 min at room temperature. The oocytes were washed with PBT. After blocking with 3% bovine serum albumin (BSA)‐PBT for 30 min, the oocytes were incubated with primary antibodies at 4°C overnight. Oocytes were washed with 3% BSA‐PBT and then incubated with secondary antibodies and 20 μg/mL Hoechst33342 for at least 2 h. Oocytes were washed and imaged using Zeiss LSM780. For immunostaining H4, oocytes were fixed with 1.6% formaldehyde in 100 mM PIPES with 1 mM MgCl_2_ and 0.1% Triton‐X100 for 30 min. The oocytes were washed with PBT and fixed again with cold methanol for 15 min.

The following primary antibodies were used: a mouse anti‐H3 antibody (1:500, Abcam, ab1791), a mouse anti‐H3.1/3.2 antibody (1:100, Active Motif), a mouse anti‐H3.3 (1:20000, Active Motif), and rabbit anti‐H4 antibody (1:200, no. 13919, Cell Signaling). Alexa Fluor 555 goat anti‐mouse IgG (H + L) (A21424) (1:500, Thermo Fisher) was used as a secondary antibody.

### 4.7 Quantification of fluorescence signal intensity

Hoechst33342 signals in z‐stack images were subjected to segmentation of whole chromosome regions using the automatic 3D surface rendering of Imaris software (Oxford Instruments). The mean fluorescence intensity of a protein of interest, as well as that of Hoechst33342 as a reference, was obtained within the segmented regions. The mean background intensity for each channel was obtained in cytoplasmic regions using Fiji (https://fiji.sc/). In each channel, the background values were subtracted from the values of the segmented regions. The ratio of the value obtained from the channel for a protein of interest to that of Hoechst33342 was calculated.

### 4.8 Live imaging

Messenger RNAs were transcribed using mMESSAGE mMACHINE T7 Kit (Ambion). The following RNAs were introduced into oocytes through microinjection: 0.04–0.6 pg H2B‐mCherry, 0.2–0.4 pg major‐satellite‐TALE‐mClover, and 0.2–0.7 pg 2mNeonGreen‐CENP‐C. Live imaging was performed using a Zeiss LSM710 or LSM780 confocal microscope equipped with a GaAsP detector and a ×40 C‐Apochromat 1.2NA water immersion objective lens, controlled by AutoFocusScreen (Rabut & Ellenberg, 2004) and MyPiC (Politi et al., 2018). For chromosome and kinetochore imaging, 17 confocal *z*‐sections (every 1.5 μm) of 256 × 256 pixel *xy* images were acquired every 3 min. Images were visualized in 3D with Imaris (Bitplane) to detect premature chromosome separation and categorize anaphase segregation.

### 4.9 Statistical analysis

Statistical significance was examined by GraphPad Prism or R. Statistical tests, sample sizes, and *P*‐values are shown in figures and figure legends.

## AUTHOR CONTRIBUTIONS

Masashi Mori and Tomoya S. Kitajima conceived the project. Masashi Mori performed experiments and analyses in the initial stage of the project, including those for Figures 1b and S1A,B. Manami Koshiguchi performed experiments and analyses for Figures 4 and 5a,b, and analyzed the data for Figure S1A,B. Osamu Takenouchi performed experiments and analyzed the data for Figure 1a,c,d, S1C,D, and 3a,b. Mei A. Mukose performed experiments and analyzed the data for Figure 3c,d. Hinako M. Takase performed experiments and analyzed the data for Figure 5c,d. Tappei Mishina performed analysis for Figure 2. Hailiang Mei and Azusa Inoue performed MNase‐seq. Miho Kihara and Takaya Abe generated *Hira* and *Chaf1a* floxed mice. Tomoya S. Kitajima supervised the project and wrote the manuscript with input from all authors.

## CONFLICT OF INTEREST STATEMENT

The authors declare no competing financial interests.

## Supporting information


**Figure S1.** Chromosomal H3 but not H2A or H2B reduces with age. (A–D) Histone H2A or H2B is not reduced with age. Oocytes at MI were immunostained with anti‐H2A (A), anti‐H2B (B), anti‐H3.1/H3.2, or anti‐H3.3 and Hoechst33342. Color code with 16 colors. BDF1 (A,B) or C57BL6 (C,D) mice at 2 months old (young) and 14–22 months old (aged) were used. Intensity of immunofluorescence relative to that of Hoechst33342 is shown. Mann–Whitney test. Parentheses show the number of oocytes from 2 (A), 1 (B), or 3 (C, D) independent experiments. Scale bar, 5 μm.
**Figure S2.** Generation of a floxed *Hira* allele Diagram of the *Hira* gene locus. Closed boxes indicate exons. Triangles indicate target sites for CRISPR‐Cas9‐mediated *Loxp* (green) insertion. Arrows indicate primer positions for genotyping. The protospacer adjacent motif (PAM, blue) and guide RNA (gRNA) target (red) are indicated.
**Figure S3.** Nucleosomal profiles. (A) Nucleosomal profiles around the transcription start sites (TSSs) of all genes in MI oocytes. MI oocytes of *Hira^f/f^ Gdf9‐Cre* mice and *Hira^f/f^* mice (4 weeks old) were used in the top panel. MI oocytes of young (2 months old) and aged (18–21 months old) BDF1 mice were used in the bottom panel. For each group, two replicates shown in B were pooled for downstream analysis. MNase‐seq data analysis was performed as described in the previous study (Hu et al., 2017; Sakamoto et al., 2023). (B) For quality check of MNase‐seq analysis in (A), AA/TT/AT di‐nucleotide periodicity was assessed for each replicate sample. All samples showed a detectable 10 base pair periodicity, indicating that MNase‐seq analysis was successful.
**Figure S4.** Generation of a floxed *Chaf1a* allele. Diagram of the *Chaf1a* gene locus. Closed boxes indicate exons. Triangles indicate the sites targeted for CRISPR‐Cas9‐mediated *Loxp* (green) insertion. Arrows indicate primer positions for genotyping. The protospacer adjacent motif (PAM, blue) and guide RNA (gRNA) target (red) are indicated. The floxed region was excised by crossing the floxed allele with a *Gdf9‐Cre* mouse to obtain a null allele.
**Table S1.** Summary of sequencing read information for MNase‐seq.
