# Supplementary material for: Aging‐associated reduction of chromosomal histones in mammalian oocytes
Source: Genes Cells. 2024 Jul 23;29(10):808–19. doi: 10.1111/gtc.13146 (PMC11555632; doi:10.1111/gtc.13146)
Supplement: Supplementary file 1 — Figure S1. Chromosomal H3 but not H2A or H2B reduces with age. (A–D) Histone H2A or H2B is not reduced with age. Oocytes at MI were immunostained with anti‐H2A (A), anti‐H2B (B), anti‐H3.1/H3.2, or anti‐H3.3 and Hoechst33342. Color code with 16 colors. BDF1 (A,B) or C57BL6 (C,D) mice at 2 months old (young) and 14–22 months old (aged) were used. Intensity of immunofluorescence relative to that of Hoechst33342 is shown. Mann–Whitney test. Parentheses show the number of oocytes from 2 (A), 1 (B), or 3 (C, D) independent experiments. Scale bar, 5 μm. Figure S2. Generation of a floxed Hira allele Diagram of the Hira gene locus. Closed boxes indicate exons. Triangles indicate target sites for CRISPR‐Cas9‐mediated Loxp (green) insertion. Arrows indicate primer positions for genotyping. The protospacer adjacent motif (PAM, blue) and guide RNA (gRNA) target (red) are indicated. Figure S3. Nucleosomal profiles. (A) Nucleosomal profiles around the transcription start sites (TSSs) of all genes in MI oocytes. MI oocytes of Hiraf/f Gdf9‐Cre mice and Hiraf/f mice (4 weeks old) were used in the top panel. MI oocytes of young (2 months old) and aged (18–21 months old) BDF1 mice were used in the bottom panel. For each group, two replicates shown in B were pooled for downstream analysis. MNase‐seq data analysis was performed as described in the previous study (Hu et al., 2017; Sakamoto et al., 2023). (B) For quality check of MNase‐seq analysis in (A), AA/TT/AT di‐nucleotide periodicity was assessed for each replicate sample. All samples showed a detectable 10 base pair periodicity, indicating that MNase‐seq analysis was successful. Figure S4. Generation of a floxed Chaf1a allele. Diagram of the Chaf1a gene locus. Closed boxes indicate exons. Triangles indicate the sites targeted for CRISPR‐Cas9‐mediated Loxp (green) insertion. Arrows indicate primer positions for genotyping. The protospacer adjacent motif (PAM, blue) and guide RNA (gRNA) target (red) are indicated. The floxed region [file GTC-29-808-s001.pdf]

Supporting Information

Aging-associated reduction of chromosomal histones in mammalian oocytes

*Correspondence: Tomoya S. Kitajima (tomoya.kitajima@riken.jp)

Figure S1

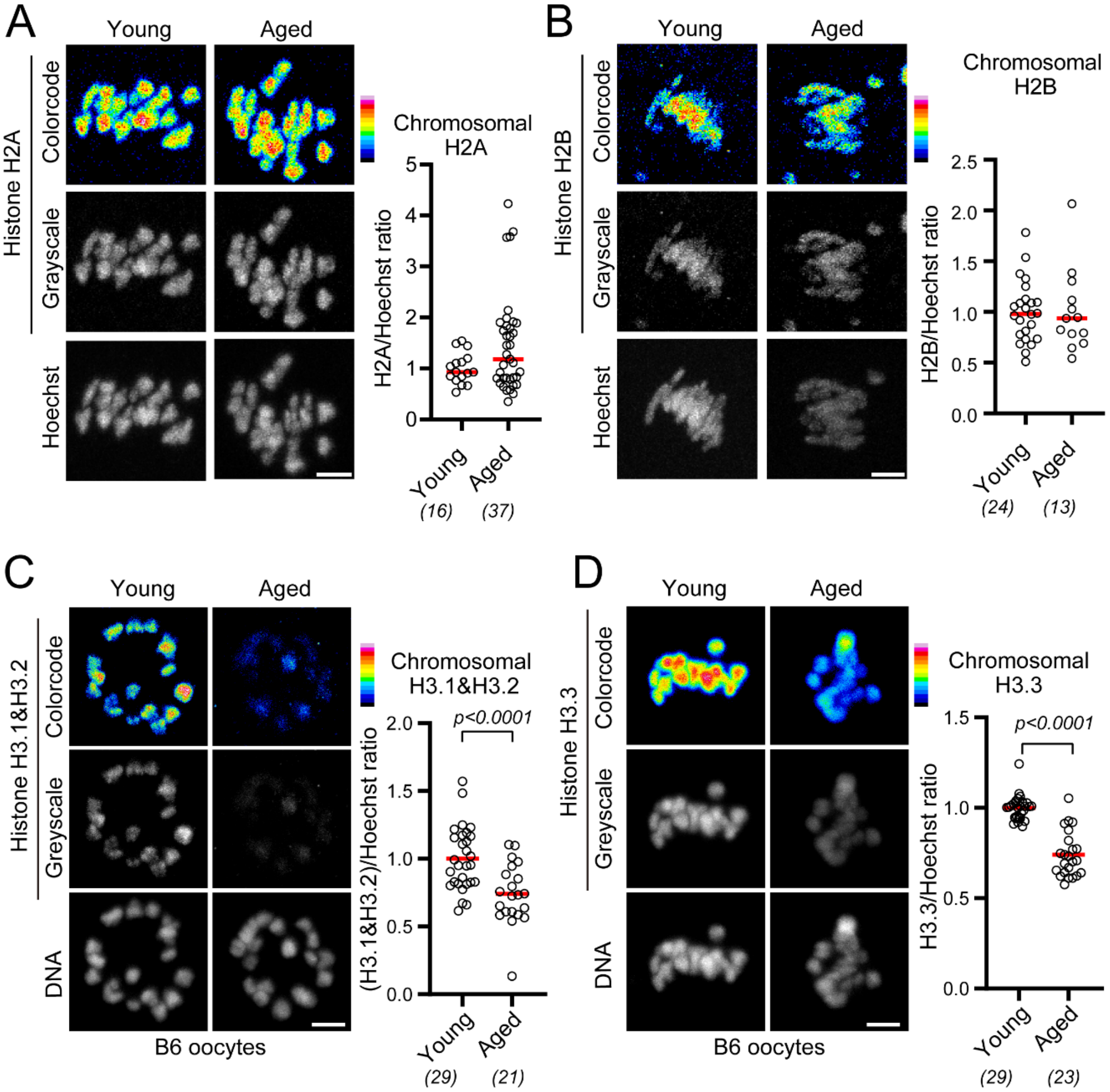

Figure S1. Chromosomal H3 but not H2A or H2B reduces with age

(A–D) Histone H2A or H2B is not reduced with age. Oocytes at MI were immunostained with anti-H2A (A), anti-H2B (B), anti-H3.1/H3.2, or anti-H3.3 and Hoechst33342. Color code with 16 colors. BDF1 (A, B) or C57BL6 (C,D) mice at 2 months old (young) and 14–22 months old (aged) were used. Intensity of immunofluorescence relative to that of Hoechst33342 is shown. Mann-Whitney test. Parentheses show the number of oocytes from 2 (A), 1 (B), or 3 (C, D) independent experiments. Scale bar, 5 μm.

Figure S2

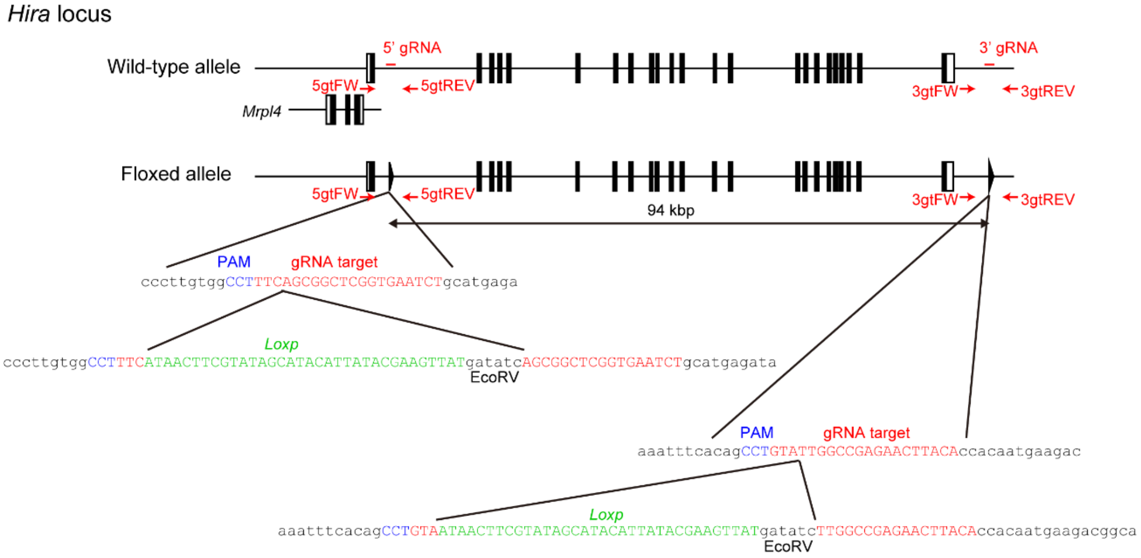

Figure S2. Generation of a floxed *Hira* allele

Diagram of the *Hira* gene locus. Closed boxes indicate exons. Triangles indicate target sites for CRISPR-Cas9-mediated *Loxp* (green) insertion. Arrows indicate primer positions for genotyping. The protospacer adjacent motif (PAM, blue) and guide RNA (gRNA) target (red) are indicated.

Figure S3

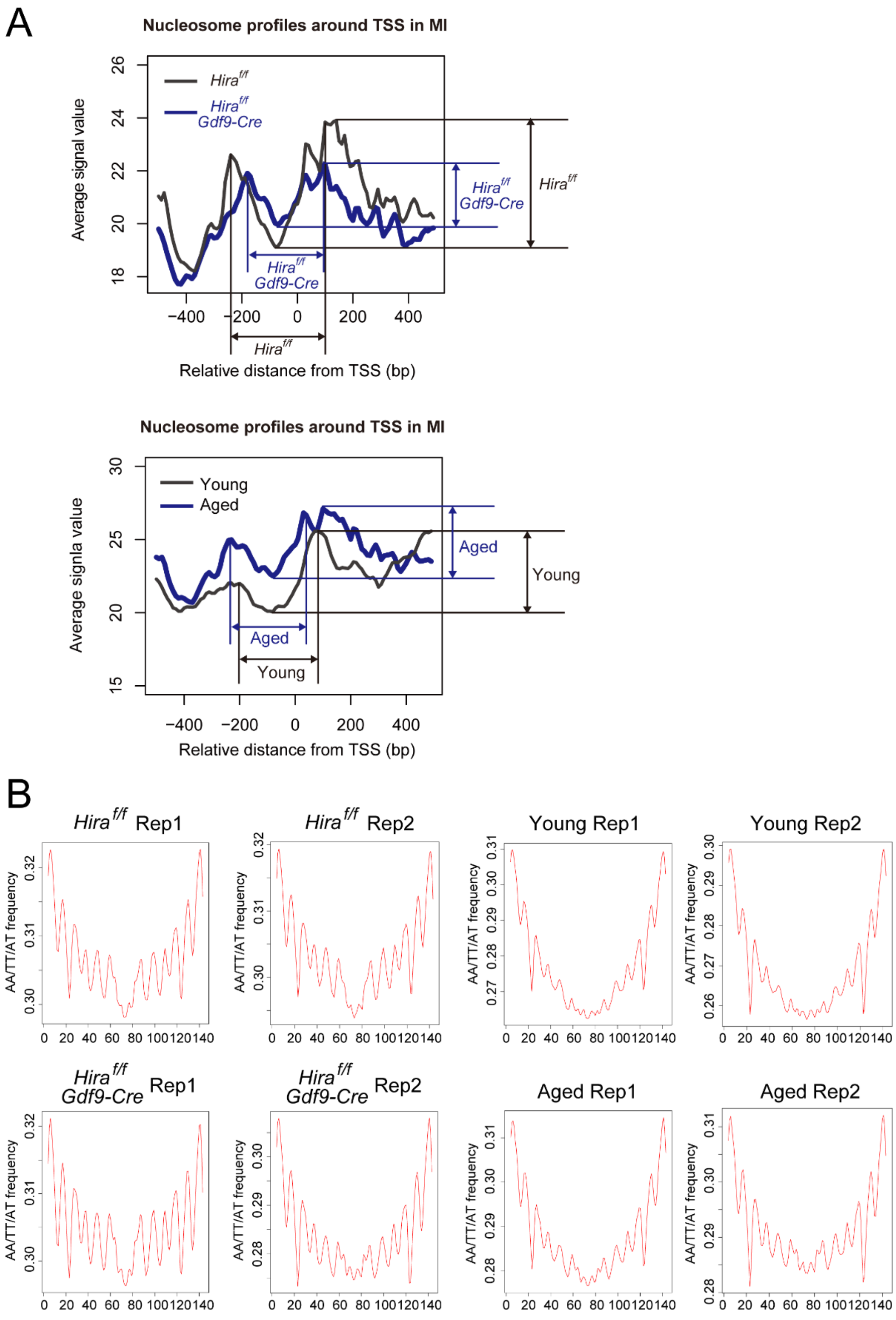

Figure S3. Nucleosomal profiles

(A) Nucleosomal profiles around the transcription start sites (TSSs) of all genes in MI oocytes. MI oocytes of *Hira*$^{f/f}$ *Gdf9-Cre* mice and *Hira*$^{f/f}$ mice (4 weeks old) were used in the top panel. MI oocytes of young (2 months old) and aged (18–21 months old) BDF1 mice

were used in the bottom panel. For each group, two replicates shown in B were pooled for downstream analysis. MNase-seq data analysis was performed as described in the previous study (Hu et al., 2017; Sakamoto et al., 2023).

(B) For quality check of MNase-seq analysis in (A), AA/TT/AT di-nucleotide periodicity was assessed for each replicate sample. All samples showed a detectable 10 base pair periodicity, indicating that MNase-seq analysis was successful.

Figure S4

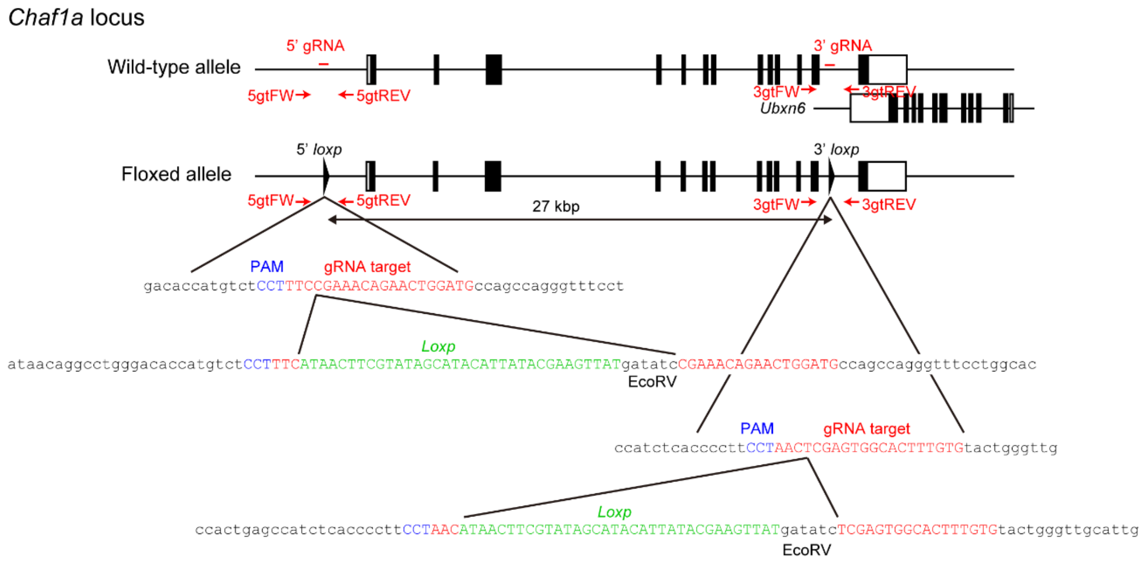

Figure S4. Generation of a floxed *Chaf1a* allele

Diagram of the *Chaf1a* gene locus. Closed boxes indicate exons. Triangles indicate the sites targeted for CRISPR-Cas9-mediated *Loxp* (green) insertion. Arrows indicate primer positions for genotyping. The protospacer adjacent motif (PAM, blue) and guide RNA (gRNA) target (red) are indicated. The floxed region was excised by crossing the floxed allele with a *Gdf9-Cre* mouse to obtain a null allele.

**Sample name**	**Raw reads**	**Useful reads**
MNase-seq_HiraWT_MI_Kitajima_Rep1_S19	48,618,248	15,059,952
MNase-seq_HiraKO_MI_Kitajima_Rep1_S20	45,946,361	14,395,986
MNase-seq_Young_MI_Kitajima_Rep1_S21	44,505,842	10,938,041
MNase-seq_Aged_MI_Kitajima_Rep1_S22	45,317,077	12,327,512
MNase-seq_HiraWT_MI_Rep2_Kitajima_S5	42,559,182	17,807,521
MNase-seq_HiraKO_MI_Rep2_Kitajima_S6	46,255,748	19,501,761
MNase-seq_Young_MI_Rep2_Kitajima_S7	51,592,767	13,926,921
MNase-seq_Aged_MI_Rep2_Kitajima_S8	46,047,992	17,740,910

Table S1. Summary of sequencing read information for MNase-seq
Raw and useful reads obtained by sequencing for MNase-seq analysis (Fig. S3) are summarized.
